# Mimetic Self-Reflexivity and Intersubjectivity in Complementary and Alternative Medicine Practices: The Mirror Neuron System in Breast Cancer Survivorship

**DOI:** 10.3389/fnint.2021.641219

**Published:** 2021-11-19

**Authors:** Vinita Agarwal

**Affiliations:** Department of Communication, Salisbury University, Salisbury, MD, United States

**Keywords:** breast cancer survivorship, complementary and alternative medicine (CAM), therapeutic relationship, intersubjectivity, mirror neuron system (MNS), self-reflexivity, mimesis, embodied simulation

## Abstract

This study examines complementary and alternative medicine (CAM) providers’ practices in the treatment of their breast cancer survivor (BCS) clients and interprets these practices within the context of existing neuroscientific research on the mirror neuron system (MNS). Purposive and snowball sampling was conducted to recruit CAM providers (*N* = 15) treating BCSs from integrative medicine centers, educational institutions, private practices, and professional medical associations across the United States. In-depth semi-structured interviewing (*N* = 252 single-spaced pages) and inductive qualitative content analysis reveal CAM therapeutic practices emphasize a diachronic form of mimetic self-reflexivity and a serendipitous form of mimetic intersubjectivity in BCS pain management to allow the providers to tune-in to their clients’ internal states over time and experience themselves as an embodied subject in an imaginative, shared space. By employing imagination and an intentional vulnerability in their embodied simulation of the others’ internal states, CAM providers co-create experiences of pain while recognizing what about the other remains an unknown. Although MNs provide the mechanism for imitation and simulation underlying empathy through a neuronally wired grasp of the other’s intentionality, the study suggests that examining mimetic self-reflexivity and intersubjectivity in the therapeutic space may allow for a shared simulation of participants’ subjective experiences of pain and potentially inform research on self-recognition and self-other discrimination as an index of self-awareness which implicates the MNS in embodied social cognition in imaginative ways.

## Introduction

Greater understanding of the prevalence, risk factors, and experience of chronic pain among breast cancer survivors (BCS) can help improve the quality of life for patients at a high risk of post-surgical pain. Research suggests that patients’ use of complementary and alternative medicine (CAM) before and after breast cancer (BC) diagnosis has been increasing (Matthews et al., 2007). A central challenge of pain management lies in the fact that the pain experienced by a subject cannot be shared by another in the same way that the subject epistemically accesses their own experience of pain. Because CAM providers employ their embodied presence in intentional, self-and other-directed healing processes (Agarwal, 2018b; Sneed and Hammer, 2018), examining their practices for conceptualization of their BCS patients’ pain can enhance understandings of embodied approaches to empathy and pain management in the therapeutic relationship. The CAM provider seeks to cultivate psychological and relational adjustments in cancer patients (Kenne Sarenmalm et al., 2013; Civilotti et al., 2015) through a range of embodied practices, including those that employ empathy (e.g., Müller et al., 2013) and intuition (e.g., Agarwal, 2018a).

Neuroscientific findings on the mirror neuron system (MNS) have examined how humans experience themselves as a subject in relationship with others (Rizzolatti and Craighero, 2004). Mirror neurons (MNs) enhance understandings of the intentional properties of interactions and help illuminate the biosocial premise of intersubjectivity in a communicative relational space (Cozolino, 2010; Gallese, 2011, 2014). The MNS mainly plays a role in understanding action, i.e., inferring the intentions of actions (Cacioppo et al., 2017). Understanding action enables learning by observing not only what another individual is doing, but also understanding the process of sense making (van Gog et al., 2009; Dobos et al., 2012; Gallagher and Bower, 2014). These findings conceptualize imitation and empathy as building blocks of human social behavior (Iacoboni, 2012). Likewise, the notion of presence in intersubjectivity brings together neurobiological and cognitive elements to understand therapeutic change as dependent upon implicit interactivity exchanges between patient and provider. As neuroscientific research on the MNS straddles the interface of bio-philosophical conceptualizations of simulation and embodiment (Gallese, 2006; Goldman, 2009), interpreting CAM provider practices in the context of MNS research can help explicate embodied phenomena such as proprioception, empathy, mentalizing, and action imitation in fields such as somatic-affective-motor studies, education, and post-structuralist critiques of human relations (e.g., di Pellegrino et al., 1992; Montero, 2006; Abraham et al., 2018; Pietrzak et al., 2018; Alcalá-López et al., 2019). Conversely, understandings of how the therapeutic relationship draws upon embodied phenomena can be enhanced by interpreting neuroscientific findings on mimicry, simulation, and intercorporeity (Girard, 1965, 1977; Gallese, 2011) in the CAM domain. However, few studies have examined the CAM therapeutic process to understand how CAM providers’ practices address BCS pain management. In this study, the author addresses the challenge of provider inaccessibility of their patient’s pain experience outside of the epistemically imprecise information afforded by the self-report to examine the experiential information provided by mimetic self-reflexivity and intersubjectivity as a potential palliative avenue. Specifically, the study examines CAM therapeutic relationship practices in BCS pain management and interprets these practices within the context of the MNS to offer conceptual insights into the CAM therapeutic relationship.

## Materials and Methods

### Participants and Procedures

**FIGURE 1:**
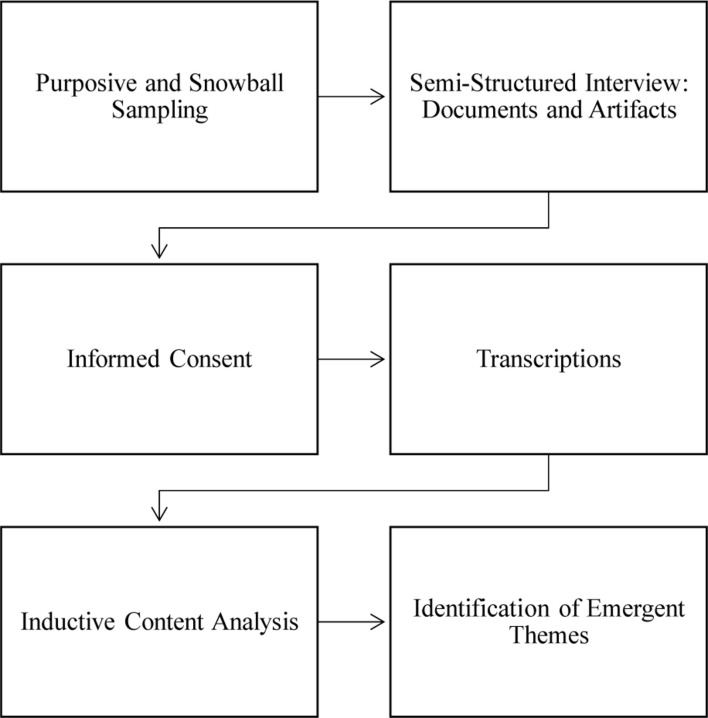
Study design.

Purposive and snowball sampling was conducted to recruit CAM providers (*N* = 15; Table 1). CAM providers were recruited through professional networks, university-based integrative medicine centers, CAM associations (e.g., chiropractic, massage, acupuncture, Ayurveda, traditional Chinese medicine, and yoga), and public searches. Inclusion criteria for participant recruitment were: based in the United States, active practitioners for the past 1 year, and have had or currently have breast cancer survivors as clients. Exclusion criteria for participant recruitment were: modalities not listed in the National Centre for Complementary and Integrative Health (NCCIH) database, and not available for an in-depth interview on Skype (without video). In-depth semi-structured interviewing (average = 45 min; 11 s; *N* = 252 single-spaced pages) and inductive content analysis were employed to examine the data (refer to Figure 1 for an overview of the study design).

**TABLE 1:** Participant characteristics*.

**#**	**Name**	**Age/race/gender**	**Practice/training**	**No. of years**	**Education**	**Practice characteristics**
1	[E]	40–49 years	Ayurveda, homeopathy, reiki	15 years	BHMS, ayurvedic wellness practitioner	Private and functional medicine group
2	[H]	50–69 years	Ayurveda	40 years	BAMS from India, masters pharmacology	Private practice
3	[D]	50–59 years	Integrative bodywork		Masters in East-West psychology, certified massage therapist	Affiliated with university
4	[N]	50–60 years	Certified yoga therapist	8 years	Certification through the IAYT yoga therapy for cancer, 500-h level	Affiliated with medical institutions
5	[A]	40–49 years	Ayurvedic practitioner, yoga therapist, licensed acupuncturist	18–19 years	BAMS, masters in acupuncture and oriental medicine, Ph.D	Affiliated with a university and private
6	[I]	40–49 years	Ayurvedic practitioner	20 years	B Science and minor chemistry, degree in massage therapy, graduate of ayurvedic institute	Private practice
7	[O]	30–39 years	Pain management using modern Eastern medicine	11 years	Master’s level training course under a mentor	Private practice
8	[K]	Age not shared	Acupuncture	20 years	Bachelor’s in biology and art and licensed acupuncture physician	Private practice
9	[C]	40–49 years	Ayurvedic medicine	19 years	Apprenticeship, ayurvedic doctors	Private practice
10	[B]	50–59 years	Licensed aesthetician, ayurvedic physician, yoga teacher	25 years	BMS	Private and affiliated with educational institutions
11	[G]	50–59 years	Training in acupuncture	18 years	3 years master’s level training and 2 years doctoral level acupuncture training	General practice with oncology referrals, family practice through a hospital system
12	[F]	50–59 years	Ayurvedic medicine	<25 years	BAMS, MD ayurvedic internal medicine, India, researcher, clinician, formulator of herbal products	Adjunct faculty and faculty at educational institutions
13	[L]	60–69 years	Scalp neuro acupuncture	<9 years	Bachelor’s in philosophy, masters in acupuncture	Private practice
14	[M]	30–39 years	Evidence-based yoga therapy, for research	15 years	Master’s in yoga therapy	Affiliated with university cancer center
15	[J]	60–70 years	Cancer and working with psychosomatic diseases	<25 years	Master’s in sound engineering, yoga therapist	Private practice, starting naturopathic oncology clinic

*National sample (United States).

**FIGURE 2:**
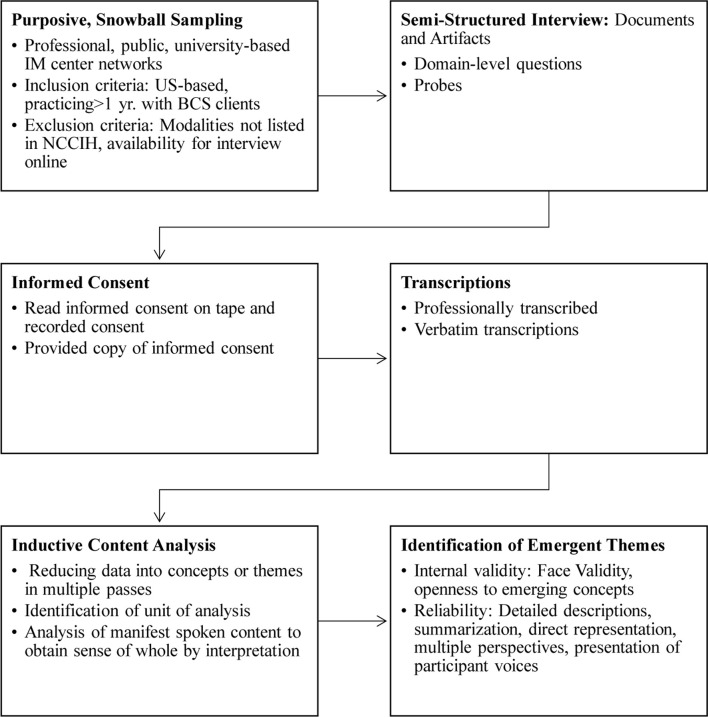
Participant recruitment, data gathering, and analytic procedures map.

Participants completed a semi-structured interview examining their communication referencing CAM therapeutic relationship factors (refer to Appendix 1 for a glossary of terms and definitions of selected humanities terms referenced in the study) adapted from existing scales comprising patient-provider communication conceptual models and dimensions (refer to Appendix 2 for domain-level questions in the interview protocol). Existing dimensions drawn from biomedical literature include the mutual constitution of goal setting for treatment, agreement on methods (task-setting) to achieve goals, healing context, congruence in worldview (e.g., pain perception), and development of a personal bond made up of reciprocal positive feelings (e.g., of confidence, trust, openness, empathy, and regard; see Appendix 2). These elements reference the biomedical therapeutic relationship premise of acceptance, following, and belief in the treatment (Stiles et al., 1998; Ardito and Rabellino, 2011). Documents and artifacts were observed where possible (e.g., intake forms, patient interview consultation questions, diagnostic procedure, and integrative healthcare policies followed at the practice), but do not form a part of this study. Provider interviews were audio-recorded (e.g., *via* Skype or phone or in-person using an audio recording app/device) and transcribed verbatim professionally (Refer to Figure 2 for the participant recruitment, data gathering, and analytic procedures map).

### Recruitment and Ethical Considerations

#### Participant Recruitment

Complementary and alternative medicine practitioners who were willing to support the study by being interviewed to describe how alternative medicine practices may help BCS patients were self-selected into the study. Participants were informed of the study focus on the communicative relationship between the provider and the patient. Providers were eligible if they had served as a practitioner for the previous year or more and had or currently had breast cancer survivors as patients. A cancer survivor was defined as a person who had received a diagnosis of cancer, from the time of diagnosis through the person’s life. Drawing upon prior work in this domain (Agarwal, 2018a,b), the in-depth semi-structured qualitative interviews assessed the preliminary constructs of trust, care, and empathy, among other concepts.

#### Ethical Considerations

Institutional review board approval (*Human Subjects Review Committee, blinded for peer review*, FWA00020237) for the study protocol (protocol # 1) was received on January 26, 2018, for the larger project with a data collection conclusion date of December 31, 2019. Informed consent was obtained through oral administration of the informed consent form to participants (audio-recorded) prior to participation in the study. Participants received a copy of the informed consent form electronically for their records (Figures 1, 2).

### Analytic Methods

**FIGURE 3:**
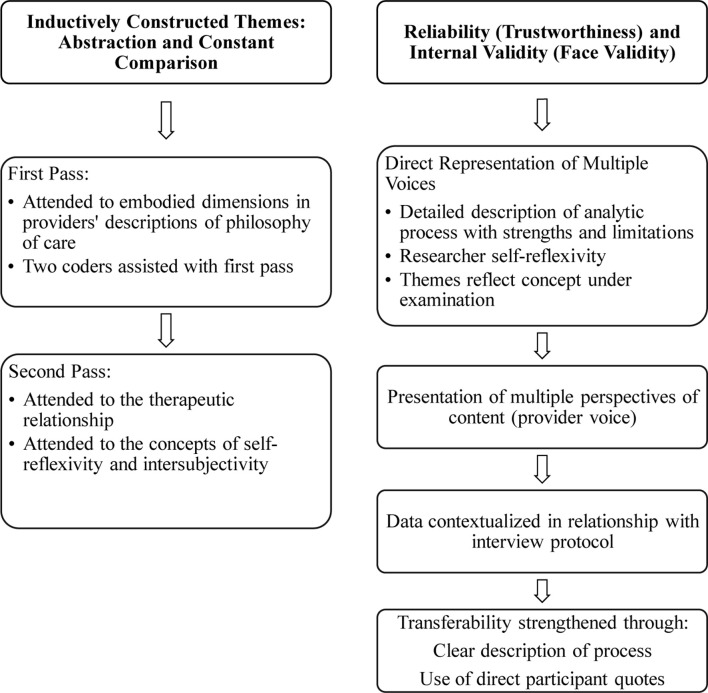
Inductive content analytic process map.

Inductive qualitative content analysis was employed for data analysis (Elo and Kyngäs, 2008; Refer to Figure 3 for a summary of the inductive content analytic procedures followed). Content analysis follows systematic and objective procedures for analyzing qualitative, textual data with a goal of enhancing conceptual understandings by reducing the data into concepts or categories that describe the phenomenon (Krippendorff, 1980; Cavanagh, 1997). Inductive content analysis moves from the specific to the general as the categories and themes are derived from the data. It has been critiqued for being ambiguous, non-generalizable, and prone to over-interpretation; however, this method offers several advantages for the present study context. First, this approach offers a rigorous inductive method that stays close to the available data for analyzing a multifaceted and under-examined phenomena (Elo and Kyngäs, 2008), such as that exemplified by the present study. Second, the limitations of inductive content analytic study findings can addressed in future studies that extend the findings through generalizable and replicable deductive analytic methods employing quantitative data. Third, by staying close to the participant voices, connecting interpretation explicitly with the description, and reducing data into categories and themes, inductive content analytic procedures can yield critical conceptual insights into the meaning making processes underlying complex communicative phenomenon. As the present study was primarily analyzed by the author, these steps also help mitigate the presence of researcher bias. Given that the study sought to provide insights into the under-examined therapeutic relational context in BCS pain management domain, the analytic process focused upon conceptual interpretation directed toward understanding “what is going on [to] obtain a sense of the whole” (Elo and Kyngäs, 2008, p. 109).

The first pass of the transcripts examined practices conceptualizing the embodied dimensions in the providers’ description of their philosophy of care. In the data preparation phase, the concepts of self-reflexivity and intersubjectivity were identified as the unit of analysis. An undergraduate student from philosophy independently read and coded 2 of the 15 transcripts (about 15% of the data) to support interrater agreement during the data clean-up process and to prepare the data for further analysis in future studies. The data analysis focused on manifest spoken content. To formulate themes in the inductive content analytic method, the researcher “comes to a decision, through interpretation, as to which [instances] to put in the same [theme]” (Dey, 1993; Elo and Kyngäs, 2008, p. 111). Inductive content analysis was performed through open coding for reducing data into categories. Memos of primary concepts in provider discourse were made (see Table 2). In the second pass, close attention was paid to the construction of categories reflecting the therapeutic relationship, self-reflexivity, and awareness of the other. Here, the goal was not simply to bring together related observations and categories, but to also distinguish these from emerging themes. To formulate the conceptual themes, in parsing second level analytical, axial codes, the “patterns, insights, and concepts” (Yin, 2018, p. 167) emerging in the descriptive level were identified to interpret relationships in the data. Abstraction and constant comparison (Creswell, 2013) were employed to collapse these. The themes were arrived at through a process of abstraction formulating a broad description of the phenomenon of inquiry and labeled using content-descriptive words.

**TABLE 2:** Complementary and alternative medicine (CAM) practitioners’ identification of goals and barriers for breast cancer survivor (BCS) clients.

**Provider name**	**Most important benefit for breast cancer survivor clients^1^**	**Most important barrier in breast cancer survivor client treatment^2^**
[E]	Assurance against relapse	Trapped in life situations
[H]	Lifestyle counseling for anxiety and hope	Emotional barriers, not knowing body
[D]	Para sympathetic nervous system activation	Not covered by insurance
[N]	Symptom relief from medication or chemotherapy	Lack of consistency in understanding yoga therapy
[A]	Quality of life	Lack of licensure for Ayurveda
[I]	Self-love, acceptance	Lack of self-care
[O]	Return to normalcy	Fear of return of symptoms
[K]	Activate healing in body, getting lymph system working	Fear will not be able to heal
[C]	Aware and empowered about wellbeing	Lack of compliance and adherence
[B]	Emotional support	Fear and inability to trust
[G]	Quality of life, improved mental and emotional state	Lack of coverage from medicare and medicaid
[F]	Emotional balance and recalibration	Fear of adverse drug/herb interactions, CAM treatment interaction with post-cancer treatments
[L]	Positive self-image	Insecure, fear of not being held or cared for
[M]	Quality of life	Patient hesitant to approach the practice itself
[J]	Moving from victimhood to empowerment (tension, depression, anger)	Cost

*^1^Benefit: most important benefit sought by yoga therapist for breast cancer survivor patients.*

*^2^Barrier: most important barrier identified by yoga therapist in breast cancer survivor patients’ treatment.*

### Reliability and Validity

To minimize bias and to enhance trustworthiness, direct representation and multiple perspectives of the content (e.g., provider voice) and its relationship with the interview protocol are presented (Creswell, 2013; Appendix 2). Trustworthiness in the inductive qualitative data analytic process was strengthened through describing the analytic process and the findings in detail that could provide the reader with a clear understanding of how the analysis was carried out and its strengths and limitations. The results section, therefore, describes the contents of the themes in sufficient detail as to explicate its conceptual meaning within the context of the study. Thus, the themes are empirically anchored in direct participant voices (Dey, 1993). The content analytic procedure requires the researcher analyze and reduce the data in themes that reflect the concept under examination in the study in a reliable manner (Elo and Kyngäs, 2008). To reduce bias in the data analysis, it was important to maintain self-reflexivity through awareness of the positionality of the participants (i.e., between the researcher-interviewer and the participant-interviewee) during the interview and data analytic process. Several concepts had culturally and philosophically specific meanings; thus, to maintain accuracy of representation and interpretation, the analytic narrative was balanced with interpretation allowing the study to connect the providers’ description of their therapeutic relationship practices with BCS care.

Reliability of the data analytic procedures in an inductive content analysis in this qualitative study is defended by providing clear descriptions linking the themes and the data through detailed accounting of the analytic process and the instances comprising the themes and presenting the relationship between data and the results in appendices and tables (see Appendices 1, 2 and Figures 1–3 and Tables 2, 3). In the results presentation process, a limitation of the qualitative content process is the challenges presented in describing actions and insights in objective, quantifiable ways (refer to Appendix 1 for a glossary of definitions of selected humanities terms referenced in the study). Although the generalizability and replicability of inductive qualitative analytic studies is severely limited, transferability of the findings may be facilitated through clear descriptions of the context, the selection and characteristics of the participants, and the data collection and analytic process. Such detailed description can support reliability of the findings and the subjective interpretations teased out in the study to enable others to follow the process and procedures of the inquiry (Elo and Kyngäs, 2008). Trustworthiness of the analysis is also enhanced through the use of participant direct quotes which “point out to readers from where or from what kinds of original data [the themes were] formulated” (Patton, 1990; Elo and Kyngäs, 2008, p. 112). In addition, in following the above data analytic procedures, participant identity was preserved in the presentation of their quotes in the findings. Toward this end, in the section “Results,” although the study preserves their mode of speaking in the writing and presentation process, some very minor corrections for syntax or missing words that were made are presented in parentheses, and CAM providers are referenced only by alphabetical letters in parentheses, e.g., [A] or [C].

**TABLE 3:** Mimetic self-reflexivity and mimetic intersubjectivity themes.

**Mimetic self-reflexivity**	**Mimetic intersubjectivity**
• Being open to intentional ways of attuning themselves to their clients’ internal states and experiences	• Providers employ embodied simulation to facilitate identification and experience themselves as a subject in relationship with another
• Willingness to be vulnerable to observing and sharing in another’s suffering	• Providers communicate their efforts to relate and identify with their patients through an empathetic presence that emphasized shared meanings
• Creating a therapeutic space of provider-patient relationality over time drawing upon precognitive states	• Providers seek to understand the interdependent space between the self and the other in intercorporeal terms emphasizing body sensations and emotions
• Simulating an embodied awareness of another’s experiences in pre-discursive and immediate ways	• Seek to identify that which is known and that which is unknown using imagination and embodied simulation
• Employing practices to build a trusting bond over time through provider’s embodied self-reflexive presence in a shared therapeutic space	• Intersubjective space of shared desire and object through meaning making using imagination to connect with possibilities

As the qualitative analytic process is subjective and interpretive, internal validity of the inductive qualitative content analysis was assessed through face validity. In order to strengthen the face validity of the findings, wherever possible, the researcher examined the data for alternative interpretations of the content (Elo and Kyngäs, 2008). As qualitative, narrative data is often critiqued as not being linear, whereby different sections may interconnect, contain overlapping multiple themes, or similar themes may be referenced in multiple ways across the data, a tolerance for uncertainty is recommended (Glaser and Strauss, 1967). Likewise, in the data analytic process, researcher openness to emerging concepts and themes was followed, while keeping the overall research inquiry in mind, in order to fully explicate the conceptual domain.

## Results

### Mimetic Self-Reflexivity

The CAM provider’s practice is concerned with being open in intentional ways to attuning themselves to their clients’ internal states and experiences (Refer to Table 3 for a summary of the mimetic self-reflexivity domains). In provider discourse, openness is illustrated as a form of vulnerability, i.e., a willingness on the part of the provider to be open to sharing the pain of the other, in this case, their clients. By opening themselves to a vulnerable form of sharing another’s suffering, the CAM provider seeks to create a therapeutic space that is aware of the relationality constituting both the provider’s and the clients’ inner experiences. In other words, over the course of each meeting and of the interactions over time, provider discourse employs their own vulnerability to pain and openness to the suffering of the other to support their clients’ sense making through embodied simulation, visualization, and observation.

Patients were often in the later stages (i.e., in stage 3 or later) of their diagnosis when they first met their CAM provider. [A] focused on helping her patients “understand they are not alone. [Together] we can fight it”; an approach which emphasizes an empathetic relational space where observation and simulation can be authentically experienced and sense making can be intentionally guided. Through communicating the healing intentions of her actions by her empathetic self-reflexive presence, [A] sought to simulate an empowering emotional space to help her clients: “reach into the state of mind where you can accept everything…[So] they feel happiness and they feel frightened.” The CAM provider drew upon self-reflexivity through language, relationality, the body, the other, and the self (Arbib, 2011) that simulated an embodied awareness of the other’s experiences in pre-discursive and immediate ways. Notably, the provider’s discursive strategies helped their clients accept and acknowledge a range of emotions (e.g., from happiness to fear).

The self-reflexive constitution of openness cultivated body acceptance in the clients through an empathetic presence, one characterized by the provider’s self-reflexive openness to being vulnerable in a shared space, but also willing to accept the other. For instance, [A’s] description illustrates how she “listen[s] when they cry, I tell them it’s okay…I hold their hand sometimes. I look at them, and their eyes…I keep quiet, let them talk.” Over time, the embodied self-reflexive practices simulated the CAM provider’s experiences in the relational space. The CAM provider’s self-reflexive simulation of her experiences while being open to her client’s vulnerability allowed her clients to acknowledge their own relations with the experiences of their body and accept the provider’s guidance to connect their emotions with their bodies.

[C], a CAM provider, described herself as “someone who shares pretty openly with my clients…And sometimes there’s an emotional connection.” Likewise, [C] noted how her clients who come to her after a procedure, such as “after chemo or radiation, people can feel kind of heavy or depressed and just go home and not really reach out that much for support…sometimes not even caring, kind of genuinely having depression.” [C’s] approach was intentionally empathetic and attuned to her clients’ experiences through her therapeutic practices, such as the Ayurveda Panchakarma, where: “we’re building a deeper bond over a period of days.” [C] describes a typical scene of sharing space with her client, when the “assistant and I are standing on the massage table holding the patient’s hands in both of our hands, and about to start the session and acknowledging something, and then we’re all three having tears.” For [C]: “that kind of context…it’s a process…everybody is kind of aligned and in-sync together.” The diachronic process of building trust over time was conducive for deepening the link between observations (e.g., of holding hands), and cultivating an internal representation of togetherness through the provider’s embodied self-reflexive presence in a shared therapeutic space.

The cultivation of trust through the practices sustains the CAM provider’s self-reflexive embodied presence. [D], when asked what was the most important benefit she sought for her clients through her practice, responded with: “pure sympathetic nervous system activation.” For [D], “the physical work” included communicating self-reflexive awareness of the client’s emotional space and simulating its connection with the body. Thus, if in her massage practice, with a particular hold, “there was an emotional response to that, or kinda the acknowledgment of fatigue that’s setting in,” [D] was responsive through the practice, and “might do more gentle holds to an area.” [D’s] embodied self-reflexive awareness of her own presence facilitated her ability to intentionally open up to and tune in to the physiological and emotional experiences of her clients: “for example, the left breast, if that was radiated, or there was surgery there, I might hold that area a little bit longer.” The hold is more than a material act; it is a communicative act simulating the process of healing over time through the relational space that communicates acceptance: “Yes, I heard that the left side is having pain right now. We don’t have to rush through this. We can just acknowledge it for a moment.” Here, the provider’s self-reflexive presence embodies and simulates the body’s experiences to support her clients in their acceptance and acknowledgment of pain.

The CAM providers’ inference of intentions to shape their client’s internal states drew upon precognitive states of their interaction. [D] said: “if you put the mind at ease by acknowledging what’s happening, by normalizing to the extent that you can, by really hearing what’s currently up for the patient mentally…then the[ir] body can relax more fully.” In this instance, through their empathetic presence, the CAM provider sought to address multiple processes—of opening up her client’s mind through acceptance, of “normalizing” their experience, and of understanding “what’s up for the patient mentally.” This intentional, self-aware opening up of the therapeutic space enabled relaxation, and ultimately, transformation through a meaningful relational connection: “And then, when you talk to the body with the hands…by the gentle hold or the acknowledgment of, yeah, I know, I remember you said, ‘This is where it hurts,’ then the body feels heard as well.” [D] said, “when the two are in conjunction, the whole person is being heard. Then, that’s when…people really respond, and the physical and emotional response is complete. It’s connected.” The diachronic cultivation of trust through the provider’s self-reflexive awareness employed empathy, simulation, and embodied vulnerability to another’s subjective experience of pain (Table 3).

### Mimetic Intersubjectivity

Complementary and alternative medicine providers’ discourse suggests they employ embodied simulation to experience themselves as a subject in relationship with others (Refer to Table 3 for a summary of mimetic intersubjectivity domains). Although [E’s] breast cancer clients come to her as survivors, she recognizes that “it’s very important that we recognize whether or not that feeling of fear of cancer is still prevailing in their mind and in their emotional aspect.” The therapeutic relationship, for [E], emphasizes: “that they have someone they can relate to, they can trust, and they feel confidence that this person might just help me come out of my situation and the feeling.” The BCS patients entering the CAM practice do not see strength or hope in their own capacity to speak to their body and relate to its changing physicality through the course of managing the disease and its treatment. In the interaction, [E] creates identification through empathy and presence, recognizing that: “already, they’ve gone through a lot in their chemotherapy…to get them free of cancer…to connect with past fears and rejections to be in the present.” [E] acknowledges this by starting not just at the time of her patients’ cancer diagnosis but by crafting a connection to the present *via* imagination with their past fears. Here the intersubjective space of the therapeutic relationship emphasized the perceptual, reflexive awareness of the self and the other to support the provider’s tuning-in the subject to shared meanings of the other’s life that resonate with the patient. For instance, one of [E’s] patients “recalled moments where she felt she was in her cradle, in her crib, and it was very dark room, and she felt lonely out there.” As [E] described, for her BCS patient, “those are past hurts for her. Any such situations as she progressed in her life…[these] situations were in her emotions, which were feelings of abandonment.” The provider’s recognition of her client’s internal fears was communicated in her efforts to relate with her clients through practices emphasizing her empathetic presence.

The CAM provider’s discourse suggests they employ their embodied experience to understand the interdependent space between the self and the other in intercorporeal terms. [F] describes how he sought to bring about a: “kind of rebirth and rebuild…finding a different purpose and meaning from this condition.” In this instance, the provider who sought to connect the patient’s body in pain with a sense of purpose and meaning through the physicality of their practice, evokes an act of creation and imagination. The CAM provider seeks to identify that which is unknown about their client’s lived experiences to arrive at a mutually empowering therapeutic relational space. Imagination of a healing space through shared co-experiencing offers the potential to create the unexpected from what is not present in the intersubjective space. For [F]: “We are not only working with the fear of cancer but [with the goal of] improving, and one of the commonest compliments that I get is, ’now I realize how good I can feel even though I had breast cancer.” Coupling imagination with an intersubjective healing space helped evoke for the clients an ability to draw upon empowering ways of visualizing themselves. Here, the CAM provider seeks to create a therapeutic relationship that can be transformational and construct individual purpose and meaning making through communication. [F] said: “eye contact is important, the body language is important, the compassion in your voice is important, you should not be just empathetic or sympathetic to their needs, but [focus on] making them feel empowered.” Empathy to body sensations and emotions can inform the provider’s ability to bring about therapeutic change through intentional interactivity exchanges with the client. [F] explains: “I’m listening to the emotions behind their words. I’m looking at their pauses, gaps, I’m looking at their body language.” Such observations shape the relationship through the provider’s intimate intersubjective knowledge of the other’s experience in the moment, by an act of imagination enjoining awareness of what is known and unknowable about the other to go beyond empathy.

In CAM provider discourse, the body and its pain is experienced as an object that is co-created through an imaginative construction in the provider’s embodied simulation of their clients’ subjective communication. [F] says: “I’m looking directly in their eyes and how they’re saying it, why they’re saying it, and more importantly, I’m also feeling the changes that they’re having on their facial expressions, the changes, the twitches, the wateriness in their eyes.” Embodied simulation of these observations permits the CAM provider to co-experience their client’s pain. For [F], they imply that “Most of the time it is a process that is very painful and difficult, [the BCS experience] has created some challenges in some ways, and they’re feeling burnt out, exhausted and tired, and they are depressed, they are unhappy, they’re angry, and they’re sad. That space and time and your ability to listen into that thing is very, very, very important” ([F]). The shared intersubjective experience of the other’s emotional space to identify their agency is supported by the CAM provider’s ability to be open to the presence of the other.

The intersubjective space sustains a shared intentional object and shared desire. [D] described how: “building that rapport and…listening both to the mind and the body feeds the trust…with [t]rust, [t]here’s confidence.” Intersubjectivity supports meaning making within the context of the jointly sustained interaction that is experienced distinctly by the provider and the client and constructs unique, but shared meanings for each. As [D] says: “So, if they didn’t trust me or want me as part of their care team, then they’re going to be on guard, and they’re not going to have the confidence that they’re in the right hands.” The provider’s empathy to body sensations and emotions informs their ability to employ imagination as a mechanism in bringing about therapeutic change. [F] acknowledges how: “where you really create this connection where they feel heard, they feel listened [to], they feel as if someone knows them really inside out because you’ve look[ed] at their life on the spectrum scale.” Imagination connects the provider’s knowledge of the other’s present with what they are becoming and in opening up possibilities that did not exist in their past state. [F] said: “This disease has literally changed them. It has shaken their belief system. It has shaken the way of their life. It has shaken their ego…It has shaken their arrogance” [F].

Complementary and alternative medicine providers’ conceptualization of intersubjectivity as an emergent space of co-experienced imagination is grounded in being present in acknowledgment and recognition of the moment. As [D] elaborates: “holding space is something that is compassionate. Acknowledging the emotions and the physical feelings of what a client is presenting is compassion.” Through rapport, listening, empathetic presence, and acknowledging what is knowledgable and that which is unknown about the client, CAM providers employ imagination to transform and empower in the shared intersubjective therapeutic space (Table 3).

## Discussion

Complementary and alternative medicine therapeutic relationship practices employ (a) mimetic self-reflexivity and (b) mimetic intersubjectivity in BCS pain management. These practices support the CAM providers’ efforts to attune themselves to their clients’ internal states as they shift over time and to experience themselves as a subject in relationship with others. The CAM provider cultivates diachronic self-reflexivity being vulnerable in sharing the pain of the other in a therapeutic space. Their openness demonstrates self-reflexive awareness of the relationality of the provider and the clients’ internal states and experiences as they develop over time. CAM providers employ intersubjectivity through embodied simulation to experience themselves as a perceptual, reflexive subject in relationship with their clients. In doing so, the CAM provider maintains an awareness of what they know about the other and recognize what will remain unknown about the other, i.e., the client, in the therapeutic relational space to allow for the emergence of the unexpected as a transformative process. In this section, the findings are discussed first, with respect to the transformative potential of CAM practices and second, with respect to interpreting them in the context of MNS research.

Complementary and alternative medicine providers developed an embodied awareness of their relationality with the client through observation and simulation and employed it to construct an empathetic presence. Their empathetic presence enabled the CAM provider to simulate togetherness for their clients, who often feel isolated from others and alone in their experiences (e.g., of body dissociation), by employing their practices in the therapeutic relational space. Through a self-reflexive awareness of their own and their clients’ experiences, the provider sought to intentionally guide sense making for their clients using practices that connected language, relationality, and their own bodies to cultivate body acceptance and relational support. The CAM providers’ simulation of embodied awareness of their clients’ internal experiences was pre-discursive and immediate in nature, drawing not upon their clients’ explanations but upon their sense making processes guided by their intentional and vulnerable self-reflexive awareness of the other over time.

Complementary and alternative medicine providers recognized that BCS clients felt isolated, depressed, and dissociated from the body’s treatment experiences post-surgery. Through their practices, they sought to build an emotional connection by employing their willingness to be vulnerable to experiencing the other’s pain in the therapeutic relationship. CAM provider approaches cultivated vulnerability to the other by building a bond over a period of time through practices such as silence in embodying and imbuing the therapeutic relational context with care. In the CAM providers’ description, this process involved holding hands, listening without judgment, sharing a quiet acknowledgment of each other’s presence, and affective response such as tears, holding a touch on the body to communicate hearing pain, and a willingness to work over time. The practices sought to simulate the other’s experiences in diachronic, pre-discursive ways, allowing them to acknowledge and connect with their own relations with the body and supporting body acceptance. The therapeutic relationship focused on enabling their clients to cultivate trust and openness where acceptance of their body’s experiences (e.g., of fatigue or pain), became possible, and where the deep bond that brought them in alignment with each other allowed their clients to relax fully and accept vulnerability while hearing their bodies without fear or anger.

Complementary and alternative medicine providers employ their embodied simulation of the other’s experiences to shape the interdependent intercorporeal space and to identify that which is unknown about their client’s experiences to cultivate a mutually empowering therapeutic relationship. The CAM provider seeks to recognize their client’s fears (e.g., of cancer), and to create identification with their journey to help them envision their presence in the moment. The intersubjective therapeutic relationship emphasized the CAM provider’s goal of creating shared meanings of their client’s journey, encompassing their deepest memories and fears, and feeling connected with their present experience of cancer in pre-discursive ways (e.g., of being lonely in a cradle and feeling abandoned in a dark room). The intersubjective space integrated imagination with the experiential and the intercorporeal as it sought to transform, a process that CAM providers described as seeking to evoke rebirth and rebuilding. The process of transformation sought to draw upon imagination to bridge what was known and what was unknown about the client’s experience in the intersubjective space of the therapeutic relationship.

In their effort to bring about a transformative change in their clients’ experience of BCS, CAM providers employed imagination to simulate the clients’ pain in embodied ways. CAM providers highlighted building trust in the relationship and gaining their clients’ confidence through paying close attention to bodily cues such as eye contact, the clients’ spoken word and how it was spoken, and in making an effort to understand why it was spoken. CAM providers noted their efforts to imagine the changes their clients were experiencing through attending closely to their facial expressions and body cues such as wateriness of their eyes or twitching of the facial muscles and described them as central to building trust in the interaction and practices. Close observation of intercorporeal cues allows the provider to simulate their clients’ feelings of pain, exhaustion, anger, sadness, or abandonment in embodied ways. Thus, the embodied space supports shared intersubjective meaning making that offers the CAM provider and their client the opportunity to imagine transformative experiences of pain within the therapeutic relationship.

A challenge in BCS non-pharmacological pain management lies in the provider’s inability to grasp pain beyond the limitations of its representational and linguistic expressions. Embodied self-reflexivity and intersubjectivity facilitates an experiential space of the providers’ embodied presence. Functional imaging studies investigating the neural mechanisms supporting the cognitive and psychological modulation of pain identify the primary somatosensory cortex (for processing of intensity and spatial features of noxious stimuli), the anterior cingulate cortex (for pain-related affect, attention, and decision-making), and the prefrontal cortex (for working memory and emotion) as significant in the processing of pain (Coghill, 2010). The embodied communication of a sensorimotor-experiential connection exemplifies how the provider employs their presence to simulate the other’s pain experience and experiences by aligning both intercorporeal and intersubjective elements.

Mimetic self-reflexivity and intersubjectivity co-create experiences where the provider learns new and unknown pathways that do not have an equivalent in their own or the clients’ prior experiences. This evokes the Girardian and Oughourlian notion of universal mimesis as “access and attachment to the mind and being of the other…to foster the opening of intersubjective expertise…to penetrating levels of relationality and social cognition” (Garrels, 2011, 2015, p. 82). The findings suggest the provider’s mimetic processes seek to be in alignment with and open to unintended new experiences. The therapeutic relationship is based on the provider’s embodied experiencing of what is known, acknowledging what is unknown about the client, and imagining what is unknown to both the provider and the client. Thus, they highlight the need for being seen, heard, and cared for. The CAM provider’s work of constructing the mimetic intersubjective space offers ways of experiencing incorporeality (e.g., the body in pain) as an imaginative endeavor. Distinct from the mirroring processes underpinning the intentional sense making of the mental processes of another conspecific (Gallese, 2011), the mimetic self-reflexive and intersubjective space co-constructs possibilities of experiencing pain through employing diachronic and serendipitous approaches.

Tapping into mimetic self-reflexivity and intersubjectivity through the mechanism of imagination opens up the self and the other to unintended, serendipitous experiences. This process emphasizes the potential of the therapeutic relationship to access the other’s subjective experiences such as pain and address it through the self-reflexive and intersubjective relational dimensions in transformative ways. Unanticipated insights and serendipitous experiences offer a glimpse of human potential by expanding understandings of biologically deterministic neuronal architectures. In the therapeutic relationship, the provider seeks to map the “self of the other onto the self, reciprocated by the mapping of the self on the other” (Gallese, 2014, p. 7) to understand their clients’ lived bodily experiences. The embodied self-reflexive and intersubjective experiencing with the other can be seen as more than the mimetic resonance and embodied simulation of the therapeutic “we-space” (Pitts-Taylor, 2016). The CAM provider’s simulated imagination of their client’s pain supported through the MNS offers the potential to create the unexpected from allowing the client to envision what is not present in the moment.

Awareness that an empathic other shares one’s pain may modify the perception of pain. Empathy, defined as a way of “in-feeling,” allows the provider to draw the clients’ attention to their own shared experience of their pain and away from their own pain stimuli, thus decreasing the pain perception (Van Ryckeghem et al., 2011). MNs provide the mechanism for imitation and simulation that underlie empathy through a neuronally wired grasp of the other’s intentionality (Iacoboni, 2007). The findings suggest that examining the imitation paradigm through the lens of the provider’s self-reflexive presence inserts imagination into the subject-self and highlights the role of vulnerability to another’s subjective experiences in epistemically simulating their experience of pain. Studies have demonstrated that the act of imagining being in pain or in a painful situation induces similar sensorimotor responses (Lelard et al., 2013). Mimetic self-reflexivity and intersubjectivity in the therapeutic space may potentially allow for a shared simulation of participants’ subjective experiences of pain. The presence of breast cancer-related traumatic dissociative symptomatology has been ascribed to intrusion, avoidance, and hypervigilance and is characterized by a rejection of, and alienation from, the self. BCS experiences generate a sense of vulnerability and loss of control (Rodin et al., 2009; Civilotti et al., 2015) centered around anticipatory worries such as fear of recurrence. The internality of the threat may affect the meaning of cancer threat such as in terms of its perceived inescapability, which makes the experience of the disease and disease impact on the individual difficult to assess (Gurevich et al., 2002). Centering the CAM provider’s perception of each client’s body experiences recognizes how embodiment transforms subjective pain perceptions. The literature suggests that the pain experience is uniquely subjective and only partially accessible to the observer through indirect self reports, thus, any response to another’s pain is always incomplete and limited in its ability to comprehend it (Dauphinée, 2007). The therapeutic space of the CAM provider-client relationship offers insights into how self-reflexive awareness (e.g., as cultivated through meditation practices) in an intersubjective context can evoke therapeutic shift.

By examining the therapeutic space of transformative BCS experiences and interpreting the practices in the context of MNS findings, the study is among the first to consider how CAM practitioners employ self-reflexivity and intersubjectivity in BCS pain management. The study findings offer ways to situate self-other discrimination and embodied presence as it informs mimetic self-reflexivity and intersubjectivity in the therapeutic healing space. The qualitative nature of the study, based on a sample of purposively recruited self-selected participants, and inductively content analyzed solely by the primary researcher, suggests that further experimental studies are needed to test the findings for replicability and generalizability. Embodiment allows the provider to cultivate intersubjectivity, and to employ their positionality in self-reflexive ways to imbue their actions with meaning for the other’s transformative experience of pain (Agarwal, 2018a). Future studies can examine how diachronic self-reflexivity and serendipitous intersubjectivity can usefully inform research on self-recognition and self-other discrimination as an index of self-awareness which implicates the MNS in social cognition (Uddin et al., 2005, 2006, 2007) in imaginative and non-biologically deterministic ways. CAM therapeutic relationship practices may potentially offer mechanisms for understanding pain through the lens of self-reflexivity and intersubjectivity by including diachronic and the serendipitous subjective processes in integrative pain management.

## Author’s Note

An earlier version of this paper was presented at the *2021 International Communication Association Virtual Annual Convention*.

## Data Availability Statement

The raw data supporting the conclusions of this article will be made available by the authors, without undue reservation.

## Ethics Statement

The studies involving human participants were reviewed and approved by the Human Subjects Review Committee, Salisbury University, MD, United States, FWA00020237, Study Protocol #1. Written informed consent for participation was not required for this study in accordance with the national legislation and the institutional requirements.

## Author Contributions

The author confirms being the sole contributor of this work and has approved it for publication.

## Conflict of Interest

The author declares that the research was conducted in the absence of any commercial or financial relationships that could be construed as a potential conflict of interest.

## Publisher’s Note

All claims expressed in this article are solely those of the authors and do not necessarily represent those of their affiliated organizations, or those of the publisher, the editors and the reviewers. Any product that may be evaluated in this article, or claim that may be made by its manufacturer, is not guaranteed or endorsed by the publisher.

## Appendix

### Appendix 1

#### Definitions of Terms^†^

1. Chronic pain: Pain that persists for longer than 12 weeks despite medication or treatment.
2. Complementary and alternative medicine: The term for medical products, systems, and practices that are not considered part of standard medical care (NCI, 2021, Retrieved from https://www.cancer.gov/about-cancer/treatment/cam).
3. Self-reflexivity: Described in phenomenology as aconscious turning of the individual toward (him/her) self; of the act of being both the observer and the object of observation; of achieving self- or other-knowledge through embodied self/other monitoring, wherein the relation with oneself unfolds through the body by way of practices that increase awareness of sensations.
4. Intersubjectivity: The process and product of sharing experiences, knowledge, understandings, and expectations with others (Oxford Reference, 2021; Retrieved from https://www.oxfordreference.com/view/10.1093/oi/authority.20110803100008603).
5. Diachronic: Of, related to, or dealing with phenomena (as of language or culture) as they occur or change over a period of time (Merriam-Webster, 2021; Retrieved from https://www.merriam-webster.com/dictionary/diachronic).

^†^*In selecting the terms above, emphasis was given to defining the terms centered in the humanities or from a humanistic perspective*.

### Appendix 2

#### Salient Conceptual Domains From Semi-Structured Interview Protocol With Providers^†‡§^

1. Provider-patient relationship: This set of questions focuses on your therapeutic relationship with your breast cancer survivor clients.
  a. Please describe the bond you have with your breast cancer survivor clients.
  b. How does your relationship with your clients help with the client’s treatment and outcomes?
  c. How does your therapeutic relationship help in establishing the client’s confidence in the treatment?
  d. How can you describe openness in the therapeutic relationship with your breast cancer survivor clients?
  e. How do you help cultivate shared decision making with clients in your therapeutic relationship with your breast cancer survivor clients?
2. Provider characteristics: This section focuses on your traits as a provider in the therapeutic relationship with your breast cancer survivor clients.
  a. How do you try to listen to clients?
  b. How do you demonstrate caring for clients in the therapeutic relationship.
  c. How do you demonstrate empathy for clients?
  d. How important is sharing of own emotions is in the therapeutic relationship?
  e. How do you establish trust and maintain trust in the relationship?
3. Breast cancer survivorship: This section seeks to understand the challenges of treating breast cancer survivors in your practice.
  a. What are the most important challenges you have faced in treatment of breast cancer patients?
  b. What are the most common benefits your breast cancer survivor patients seek from your practice?
  c. What are your desired/ideal goals from your practice for the treatment of breast cancer survivors?
4. Intake questions:
  a. What is the kind of information you gather during intake from your new breast cancer survivor clients?
  b. How do you use the information you gather to tailor your treatment to your clients?

^†^*Demographic and participant characteristics presented in Table 1*.

^‡^*Most important benefits and barriers faced by providers for breast cancer clients presented in Table 2*.

^§^ *Existing scales reviewed from*:

Eveleigh, R.M., Muskens, E., van Ravesteign, H., van Dijk, I., van Rijswijk, E., Lucassen. (2012). An overview of 19 instruments assessing the doctor-patient relationship: different models or concepts are used. *J. Clin. Epidem.* 65, 10–15. doi: 10.1016/j.jclinepi.2011.05.011.

Ridd, M. J., Lewis, G., Peters, T. J., and Salisbury, C. (2011). Patient-doctor depth-of relationship scale: development and validation. *Ann. Fam. Med.* 9, 538–545. doi: 10.1370/afm.1322.
